# Adjusted Particle Size Eliminates the Need of Linkage of Antigen and Adjuvants for Appropriated T Cell Responses in Virus-Like Particle-Based Vaccines

**DOI:** 10.3389/fimmu.2017.00226

**Published:** 2017-03-06

**Authors:** Ariane C. Gomes, Anna Flace, Philippe Saudan, Franziska Zabel, Gustavo Cabral-Miranda, Aadil El Turabi, Vania Manolova, Martin F. Bachmann

**Affiliations:** ^1^The Jenner Institute, Oxford University, Oxford, UK; ^2^Cytos Biotechnology AG, Schlieren, Switzerland; ^3^Dermatology, University Hospital, Zürich, Switzerland; ^4^Immunology, Inselspital, Bern, Switzerland

**Keywords:** VLPs, vaccines, HPV, CpG, adjuvant

## Abstract

Since the discovery of the first virus-like particle (VLP) derived from hepatitis B virus in 1980 (1), the field has expanded substantially. Besides successful use of VLPs as safe autologous virus-targeting vaccines, the powerful immunogenicity of VLPs has been also harnessed to generate immune response against heterologous and even self-antigens (2–4). Linking adjuvants to VLPs displaying heterologous antigen ensures simultaneous delivery of all vaccine components to the same antigen-presenting cells. As a consequence, antigen-presenting cells, such as dendritic cells, will process and present the antigen displayed on VLPs while receiving costimulatory signals by the VLP-incorporated adjuvant. Similarly, antigen-specific B cells recognizing the antigen linked to the VLP are simultaneously exposed to the adjuvant. Here, we demonstrate in mice that physical association of antigen, carrier (VLPs), and adjuvant is more critical for B than T cell responses. As a model system, we used the E7 protein from human papilloma virus, which spontaneously forms oligomers with molecular weight ranging from 158 kDa to 10 MDa at an average size of 50 nm. E7 oligomers were either chemically linked or simply mixed with VLPs loaded with DNA rich in non-methylated CG motifs (CpGs), a ligand for toll-like receptor 9. E7-specific IgG responses were strongly enhanced if the antigen was linked to the VLPs. In contrast, both CD4^+^ and CD8^+^ T cell responses as well as T cell-mediated protection against tumor growth were comparable for linked and mixed antigen formulations. Therefore, our data show that B cell but not T cell responses require antigen-linkage to the carrier and adjuvant for optimal vaccination outcome.

## Introduction

Most prophylactic vaccines are designed to induce strong antibody responses, while many therapeutic vaccines against chronic viral infections and cancer aim to induce T cell responses (5, 6). However, for many vaccines currently under development, this dictum is challenged, and strong B and T cell responses are likely required to achieve protection. Thus, understanding the rules that govern induction of B versus T cell responses and identifying commonalities and differences between them represents an important goal in vaccinology and immunology.

Formulation of vaccines in adjuvants usually enhances both B and T helper (T_H_) responses (7). However, it is often unclear whether the adjuvants enhance B cell responses directly or indirectly, via enhancing follicular T_H_ cell responses. We have recently shown that the adjuvant CpGs linked to antigen enhances B cell responses by activating B cells directly through TLR9 recognition (8, 9), which required internalization of the CpG. By contrast, CpGs mixed with antigen may primarily enhance B cell responses by facilitating Th cell activation, thereby increasing antibody responses indirectly (10–12). As a general rule, it is important that the immunological target cells [dendritic cells (DCs) or B cells] are simultaneously activated by the adjuvant and exposed to the antigen (5, 13). One way to ensure co-exposure to antigen and adjuvant is the physical linkage of the two components. Something readily achieved by conjugation with covalent chemical bonds or by packaging adjuvant into liposomes or virus-like particles (VLPs). However, for good manufacturing practice production, covalent linkage might be a complicated and costly endeavor, especially if the antigen is complex or if the goal is patient-specific vaccination. Hence, a simple admixed formulation could be advantageous under these circumstances.

The trafficking of antigen from the periphery to lymph nodes (LNs) or the spleen is essential to drive T cell and B cell activation (5), and it is well established that one of the main factors governing influx to the LNs is the size of the particles (14–16). Particles in the nanometer range can flow freely within the lymph, rapidly reaching LNs where they can encounter relevant B cells and APCs that will activate CD4^+^ and CD8^+^ T cells (17–19). By simply mixing antigens and adjuvants of similar size, it may be possible to target the same individual cells within the LNs. To test this, we generated particulate adjuvants and antigens of similar size, mixed them freely before injecting the formulation into mice, then observed if they indeed were draining to the same cells within LNs. As adjuvant, we used VLPs derived from Qβ bacteriophages packaged with CpGs, having a size of 30 nm. The recombinant oncoprotein E7 derived from the human papilloma virus (HPV) was chosen as the target antigen as it forms oligomers with a size of around 50 nm (20). To test the impact of antigen size and co-drainage, we also used the immunodominant peptide E7_49–57_ derived from the E7 protein, representing a H2-D^b^-restricted CTL epitope (12). We found that covalent linkage was essential for maximal B cell for peptide and particle-based vaccine responses, regardless of the size of the antigen. In contrast, an admixed formulation of E7 oligomers with CpG-loaded VLPs was sufficient to induce optimal CD4^+^ and CD8^+^ T cell responses that proved to be protective as a therapeutic vaccine when tested in an HPV tumor model. Mixing free E7_49–57_ peptide with CpG-loaded VLPs, however, failed to induce strong T cell responses, suggesting that adjusted particle size may be sufficient to co-deliver antigen and adjuvants to the same DCs for optimal T cell induction, eliminating the requirement for the linkage of the two entities.

## Materials and Methods

### VLP and E7 Production

HPV-E7 protein containing a short C-terminal GGC linker was recombinantly expressed in *Escherichia coli*, solubilized from the inclusion body fraction by 8M urea and purified using affinity and size-exclusion chromatography. The denatured protein migrated as a 14-kDa single band in reducing SDS-PAGE (data not shown). After refolding by dilution and dialysis against NaCl-MES-containing buffer, E7 protein spontaneously formed oligomers. Qβ VLP production and purification have been described in detail elsewhere (21).

### CpG ODN and Antigen Sequence

CpGs 1668 with phosphorothioate backbone were purchased from Invivogen (sequence: 5′ tccatgacgttcctgatgct 3′). The protein and peptides E7_49–57_ were produced in a modified version with additional 3 aa (GGC) added to the C terminus E7 (Proimmune, UK) to allow coupling to VLPs. E7 protein sequence UniProt database: P03129. E7_49–57_ peptide sequence: RAHYNIVTFGGC.

### Measurement of Anti-E7 and Anti-VLP Antibodies by ELISA

Anti-VLP and anti-E7 antibody titers were measured in the serum of mice vaccinated 21 days earlier with unmodified VLP. A total of 96-well plates were coated overnight with 5 μg/mL of unmodified VLP or 5 μg/mL of E7 oligomers. After blocking for 2 h with 2% bovine serum albumin phosphate-buffered saline (PBS), serum obtained from vaccinated or control mice (diluted 1:500 to 1:12,500) was added and plates were incubated for 2 h at room temperature. After washing the plates three times with PBS-0.05% Tween, horseradish peroxidase-labeled goat anti-mouse immunoglobulin G (IgG-HRP) (Jackson ImmunoResearch, UK) was added for 1 h, followed by the addition of 3,3′,5,5′-tetramethylbenzidine (TMB) Sigma, as a substrate before reading the optical density (OD) at 450 nm (OD450). Titers are expressed as serum dilutions at the half-maximal OD (OD50).

### Association ELISA

Plates (Nunc-Immuno MaxiSorp) were coated with anti-E7 antibody, each of the vaccine preparations were added at a concentration corresponding to 60 ng/mL of E7 protein. Following washing and incubations, anti-Qβ monoclonal antibody was added followed by secondary detection antibody goat anti-mouse IgG-HRP and TMB. Data expressed in OD450.

### Vaccine Preparation

Vaccines were prepared by chemical coupling as described elsewhere (2). Purified Qβ VLPs (2 mg/mL in PBS) were derivatized by a 1-h incubation at room temperature with a 10-fold molar excess of succinimidyl-6-(β-maleimidopropionamido)hexanoate (Pierce, Rockford, IL, USA). Free cross-linker was removed by diafiltration with Amicon Ultra Centrifugal Filters, 100 kDa MWCO. Derivatized Qβ VLPs and E7_49–57_-GGC (peptide at fivefold molar excess) or E7 protein were then incubated for 3 h at room temperature to allow cross-linking. Unbound material was removed by diafiltration with Amicon Ultra centrifugal filters, 100 kDa MWCO for the peptide and size exclusion for the E7 protein. Efficiency of cross-linking was analyzed by SDS-PAGE.

### Packaging of CpG into Qβ VLPs

Performed as described elsewhere (22). Briefly, bacterial RNA trapped into VLPs during recombinant expression was digested with RNAse A (Merck) for 5 h at 37°C. RNAse-treated VLPs were combined with 120 nM/mL of CpG and incubated for further 3 h at 37°C. Excess of CpG was removed by dialysis against PBS.

### Immunization and Trafficking Experiments

C57BL/6 mice (9–12 weeks old; Harlan) were injected s.c. with 80 μg of either vaccine formulation. Blood was collected from the tail vein and serum and PBMCs separated for further analysis.

E7 protein or E7_49–57_ was labeled with AlexaFluor 488 C5-maleamide as described by the manufacturer and with Qβ labeled with AlexaFluor 647 or PE as instructed by the manufacturer (all from Thermo Fischer). C57BL/6 mice (9–12 weeks old; Harlan) were injected s.c. with 30 μg of VLP and peptide into the hind leg.

All mouse experiments have been performed in accordance with the local welfare legislation and under valid animal experimentation licenses.

### Cells and Flow Cytometry

Popliteal and inguinal LNs were isolated and a single-cell suspension was prepared by incubating LN with 1 mg/mL collagenase D (Roche) and 0.04 mg/mL DNase I (Boehringer) in 5% FSC containing DMEM, for 30 min at 37°C. Organ pieces were passed through a 70-μm cell strainer and stained for cell-specific markers. The following fluorochrome-labeled antibodies were used: CD11c, CD8a (all from eBioscience), F4/80, Live/Dead Aqua cell stain (all from Life Technologies).

Spleens were isolated smashed through a 70-μm cell strainer using the plunger of a syringe (Falcon), red blood cells were lysed with ACK solution (Lonza). The following fluorochrome-labeled antibodies were used: CD3, CD8a, Live-Dead dye, IFNγ, TNFα, and IL-17 (eBioscience).

Primary culture of DCs from LNs of a naïve mice were harvested and incubated for 24 h with 10 nM of E7_49-51_ conjugated with Alexa488 and 1 μg/mL of Qβ conjugated with Alexa647. CD11c^+^ F4/80^−^ DCs were subsequently analyzed by flow cytometry for uptake of peptide and Qβ.

### Tumor Model

Female C57BL/6 mice at age of 10–11 weeks were injected with 1.5 × 10^5^ TC-1 cells expressing HPV16 E7 oncoprotein. Eight days post injection of tumor cells, mice developed palpable tumors and were subjected to a weekly immunization schedule with E7 coupled or mixed to Qβ(1668) for 49 days. Tumor size was recorded daily and was calculated as follows: W*W*L/2, where *W* is tumor width (centimeters) and *L* is tumor length (centimeters). Mice showing signs of suffering or reaching tumor size larger than 1,000 cm^3^ were euthanized.

## Results

### Characterization of Qβ-VLPs and E7

**Figure 1:**
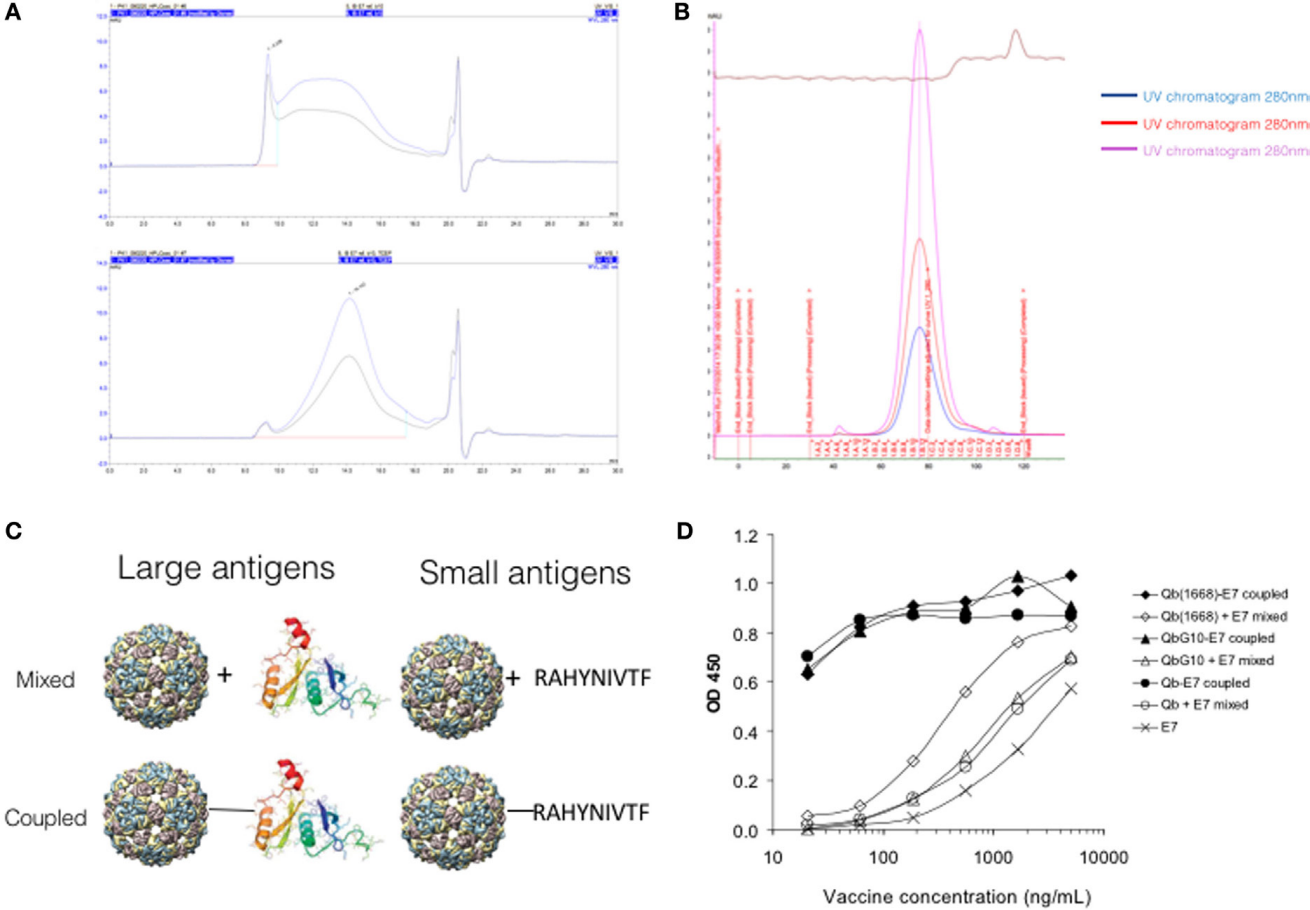
**Size characterization of Qβ virus-like particles (VLPs) and E7 protein**. **(A)** Size-exclusion chromatography of refolded E7 in buffer without reducing agent (upper panel) using TSKgel G4000SW column (resolution between 2 × 10^4^ and 7 × 10^6^ Da). Refolded E7 (bottom panel) forms oligomers with elution time between 9.3 and 15 min. **(B)** Size-exclusion of Qβ VLPs in a S500 column eluted with phosphate-buffered saline. Narrow peak shows narrow size distribution. **(C)** Schematic representation of the system of antigens. VLPs have an average size of 30 nm, E7 protein of 50 nm, and the peptide has a size of 1.7 kDa. The plus signal (+) indicates the mixed formulation of VLP and antigen, the dash (–) indicates chemical coupling of antigen and VLP. **(D)** Immunoplate assay demonstrating the absence of physical association between VLP and E7 protein in the mixed formulation. Plates were coated with anti-E7 antibody, each of the vaccines preparation was added at a concentration corresponding to 60 ng/ml E7 protein. Following washing and incubations, anti-Qβ monoclonal were added followed by detection antibody. Data expressed in optical density (OD).

Size-exclusion chromatography analysis of refolded E7 showed a wide distribution elution profile corresponding to molecular weights between 17 kDa and 10 MDa (void volume of the column, Figure 1A, upper panel). The refolded E7 was treated under reducing conditions, which narrowed the molecular weight distribution to a major peak with average size of 670 kDa (Figure 1A, bottom panel) and demonstrated the role of disulfide bonds in E7 multimerization, as described elsewhere (20). The 14 kDa capsid protein of the Qβ bacteriophage is expressed in *E. coli* and self-assembles to form a VLP with molecular weight of approximately 3 MDa. Electron microscopy and dynamic light scattering (not shown) demonstrates that Qβ VLPs are single particles with average diameter of 30 nm. The size-exclusion profile demonstrated a narrow size distribution of the VLP on a S500 column (GE) (Figure 1B). Thus, Qβ VLPs and non-reduced E7 oligomers exhibit roughly comparable sizes of 30–50 nm.

### Qβ and E7 Protein Are Physically Associated by Chemical Coupling but Not by Mixing

**Figure 2:**
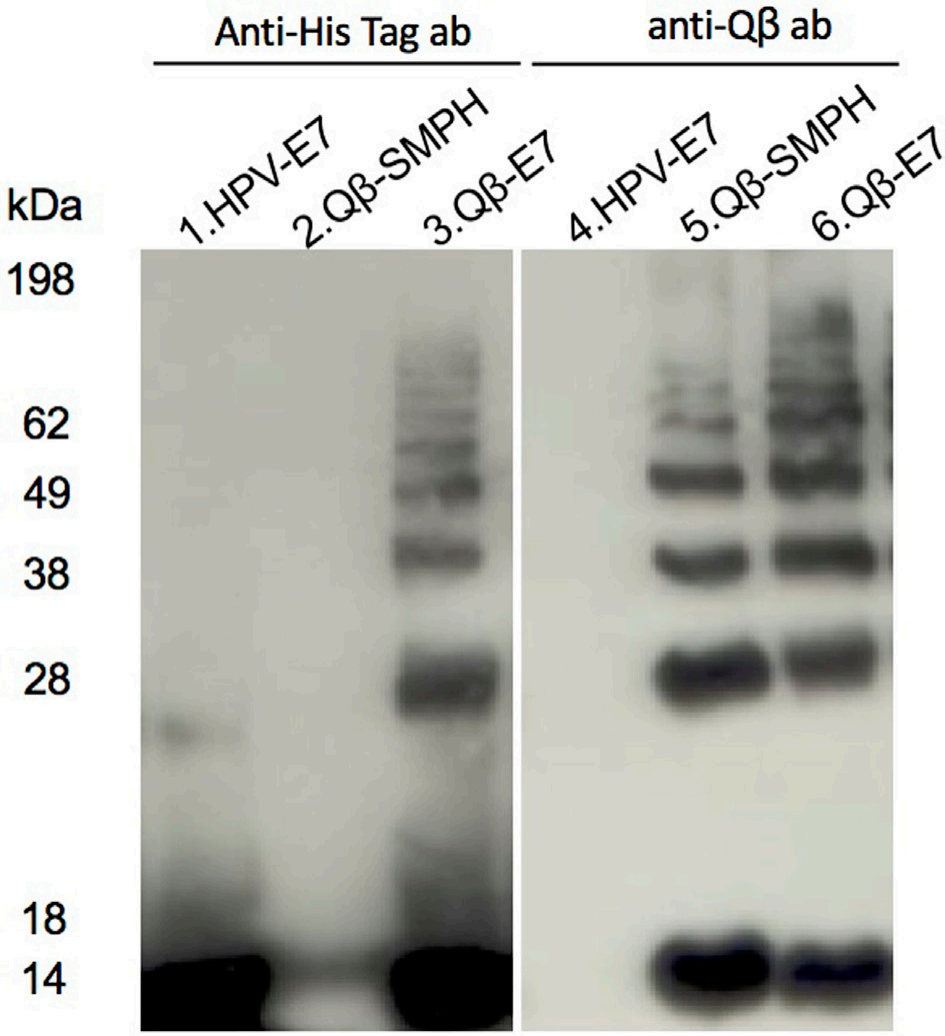
**Biochemical analysis of the coupling of Qβ and hPV.e7**. HPV.E7 (lane 1 and 4), SMPH reacted Qβ (2 and 5), and coupled Qβ-E7 (3 and 6) were separated by SDS-PAGE under reducing conditions and analyzed by Western blot. HPV-E7 was detected in lanes 1–3 by anti-His tag antibody (His Tag Ab), and lanes 4–6 were assayed with anti-Qβ specific antibody (Qβ Ab).

In order to compare the immunogenicity of mixed and covalently linked antigen and VLP, the E7 protein containing a short cysteine-containing linker (-GGC) was chemically cross-linked via the free cysteine to surface lysine on Qβ using the hetero-bi-directional cross-linker, SMPH. The efficiency of the cross-linking was assessed by separation on SDS-PAGE under reducing conditions and analyzed by Western blot (Figure 2). Alternatively, purified Qβ and E7 protein were simply mixed. The model of mixed and coupled formulations is represented in Figure 1C. To assess whether mixing would result in spontaneous association of Qβ to E7 oligomers, a sandwich ELISA assay was performed using anti-Qβ VLP capture antibodies and anti-E7 detection antibodies. The assay demonstrated that E7 was only associated with Qβ VLPs upon chemical coupling, while simple mixing did not result in any physical association (Figure 1D). To ensure that loading of VLPs with CpGs did not alter the binding properties of VLPs, the ELISA was repeated with Qβ VLPs loaded with B-type CpG 1668 (Qβ (1668)). Similarly, no association between E7 and Qβ VLPs could be observed (Figure 1D). VLPs loaded with CpG are represented as Qβ(1668), while “Qβ” will be used to indicate unmodified VLPs (naturally loaded with *E. coli*-derived ssRNA).

### Linkage of Qβ and E7 Is Not Required for Uptake by the Same DCs

**Figure 3:**
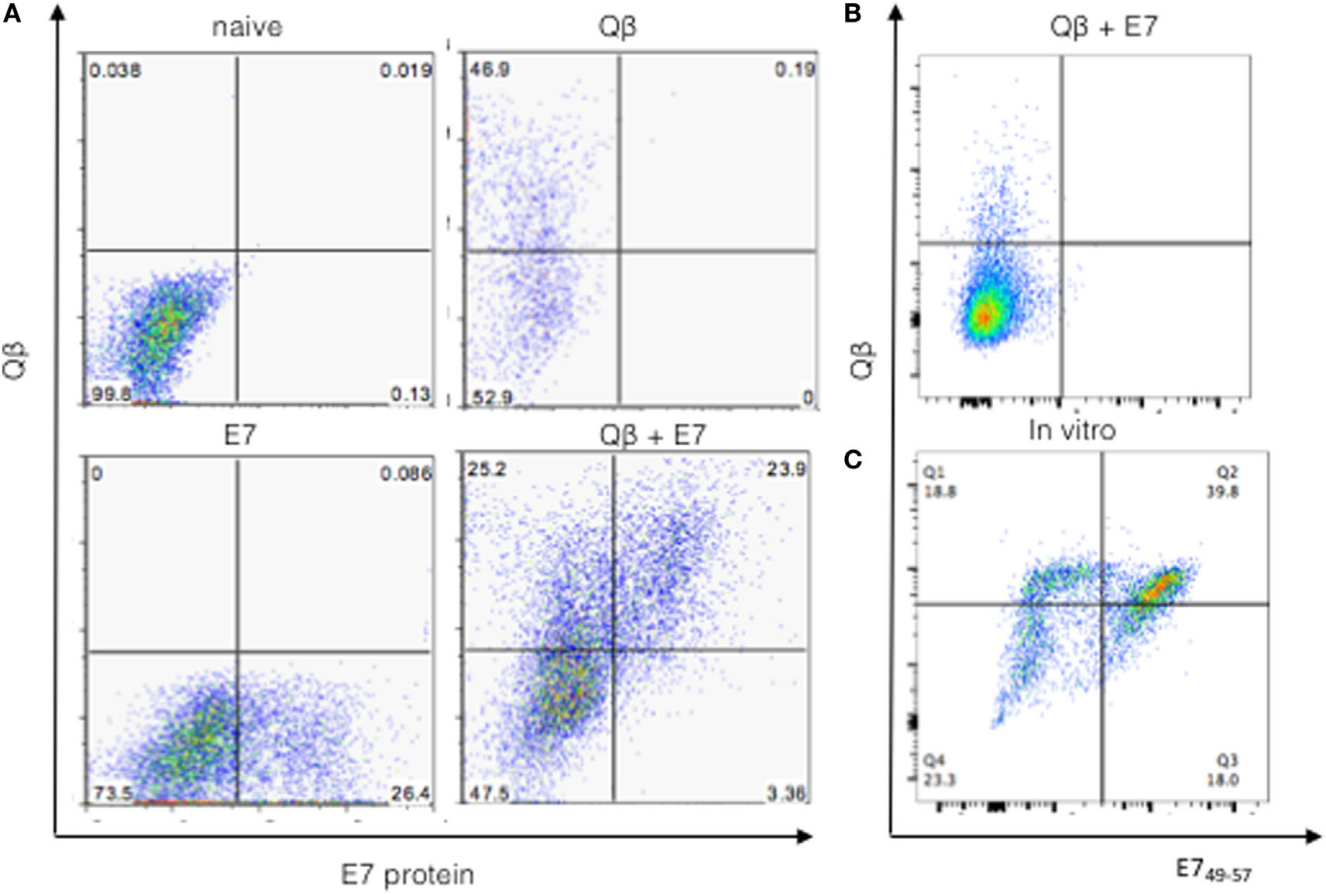
**Draining of Qβ virus-like particle (VLP) and antigens to LNs**. **(A)** Qβ-VLP mixed with E7 oligomers are taken up by the same population of DCs in the draining lymph nodes. Mice were injected either with Qβ-VLP-Alexa488 or E7-phycoerythrin (PE) or a mixture of both particles (100 μg of each). The uptake of the fluorescent proteins into DCs of the draining lymph node (popliteal) was analyzed by flow cytometry. Frequency plot is gated on DCs (CD11c^+^ F4/80^−^). **(B)** Frequency plot of cells from draining LNs 24 h postinjection with 100 μg of Qβ-Alexa647 and 100 μg of E7_49–57_ peptide labeled with Alexa Fluor 488. **(C)** *In vitro* uptake of Qβ-Alexa647 and E7_49–57_ Alexa488. Single cell preparation draining LN of naïve C57BL/6 mice was prepared and pulsed for 24 h with 1 μg/mL VLP and 10 nM of peptide. Frequency plot is gated on DCs (CD11c^+^ F4/80^−^).

Drainage of Qβ and E7 was assessed by labeling particles with Phycoerythrine (PE) and Alexa488, respectively. Flow cytometry analysis demonstrated that a subpopulation of resident DCs of draining LNs simultaneously took up both E7 and Qβ VLPs (Figure 3A). A total of 23% of cDCs (CD11c^high^F4/80^−^) from the popliteal LN were double positives for Qβ and the antigen E7-Alexa488. Thus, antigen and adjuvants, i.e., E7 and Qβ VLPs, were taken up simultaneously *in vivo* by the same DCs.

To confirm the hypothesis that antigen and VLP size was the limiting factor on antigen and VLP distribution, the E7 derived peptide H2-D^b^ E7_49–57_ was used as antigen instead of the E7 protein. Injection of free peptide E7_49–57_ labeled likewise with Alexa488 and Qβ VLPs labeled with Alexa647 did not result in simultaneous uptake by DCs in draining LNs (Figure 3B). As a matter of fact, the peptide could not be found in LNs 24 h after injection, indicating that small peptides do not drain efficiently to LNs and are not efficiently transported by DCs.

To exclude potential methodological limitations in visualizing peptide interaction with DCs by flow cytometry, an *in vitro* pulsing experiment was performed. Primary cultures of DCs from LNs of a naïve mice were incubated for 24 h with the peptide E7_49-51_ conjugated to Alexa488 and Qβ conjugated to Alexa647. CD11c^+^ F4/80^−^ DCs were subsequently analyzed by flow cytometry for uptake of peptide and Qβ. As shown in Figure 3C, almost 50% of the DCs simultaneously interacted with the peptide and Qβ, which confirmed the capacity of small peptides to interact with DCs *in vitro* and suggested that subcutaneously injected small peptides do not efficiently traffic to the LN.

### Packaging of CpG into VLPs Is Necessary to Induce T Cell Responses

**Figure 4:**
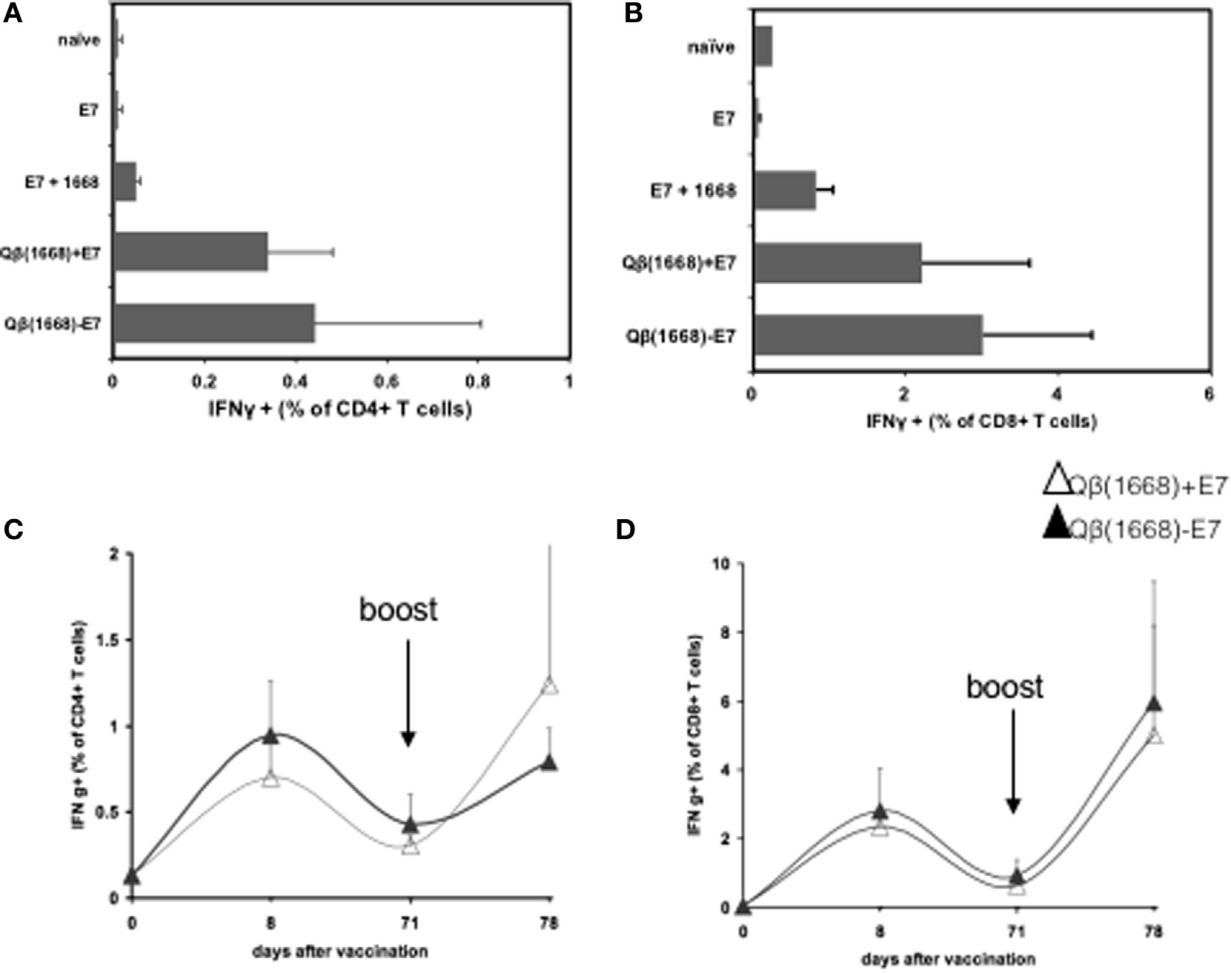
**Coupling of similar size antigens to VLPs is not required for cytokine production by T cells**. C57BL/6 mice were immunized, and T cell responses were assessed 8 days later. **(A)** IFNγ production by CD4^+^ T cells after immunization with E7 protein, E7 mixed with 1668, Qβ(1668) + E7 mixed and Qβ(1668) − E7 coupled. **(B)** IFNγ production by CD8^+^ T cells after immunization with E7 protein, E7 mixed with 1668, Qβ(1668) + E7 mixed, and Qβ(1668) − E7 coupled. Mice were immunized with mixed or coupled vaccines. Boost was administered 70 days after first dose, blood was collected on days 0, 8, 71, and 78 for IFNγ measurements. **(C)** Kinetics of IFNγ production by CD4^+^ T cells from blood. **(D)** Kinetics of IFN production by CD4^+^ T cells from blood. Data represented as mean + SD *n* = 5 mice per group.

The contribution of the vaccine components for generation of CD4^+^ and CD8^+^ T cell responses was investigated by injecting mice with E7 protein either alone or mixed with CpG and Qβ(1668) either coupled or mixed with E7. The production of IFNγ by CD4^+^ and CD8^+^ T cells was measured 8 days after vaccination. E7 alone or mixed with free CpG induced low percentage of E7-specific T cells. E7 protein mixed or coupled with Qβ(1668) efficiently induced T cells producing IFNγ demonstrating the superior T cell immunogenicity of a vaccine containing VLPs-packaged CpGs (Figures 4A,B). More importantly, levels of CD4^+^ and CD8^+^ T cell responses induced by E7 protein either mixed or coupled to Qβ(1668) were similar, suggesting that chemical association of the antigen and adjuvant is dispensable for priming (Figures 4A,B) T cell responses.

### Mixing of Antigens and CpG-Loaded VLP of Similar Size Is Sufficient for Induction of Strong T Cell Responses

After establishing that Qβ(1668) was necessary to induce IFNγ production by CD8^+^ T cells in response to E7 and that DCs simultaneously take up antigen and VLP if simply mixed, it was investigated whether such formulation was sufficient for induction of protective T cell responses or whether covalent linkage of E7 oligomers to Qβ(1668) VLPs was necessary. First, the kinetics and duration of the response were measured. E7 was either chemically coupled to or mixed with Qβ(1668) and C57BL/6 mice were immunized s.c. in a prime-boost scheme 70 days apart. T cell responses were followed in the blood by intracellular cytokine staining for IFNγ. Both experimental groups mounted strong primary CD4^+^ and CD8^+^ T cell responses that had declined by day 70 and were strongly increased after the boost injection (Figures 4C,D, respectively). CD4^+^ and CD8^+^ T cells from the blood were analyzed by intracellular cytokine staining upon *in vitro* stimulation on day 78.

**Figure 5:**
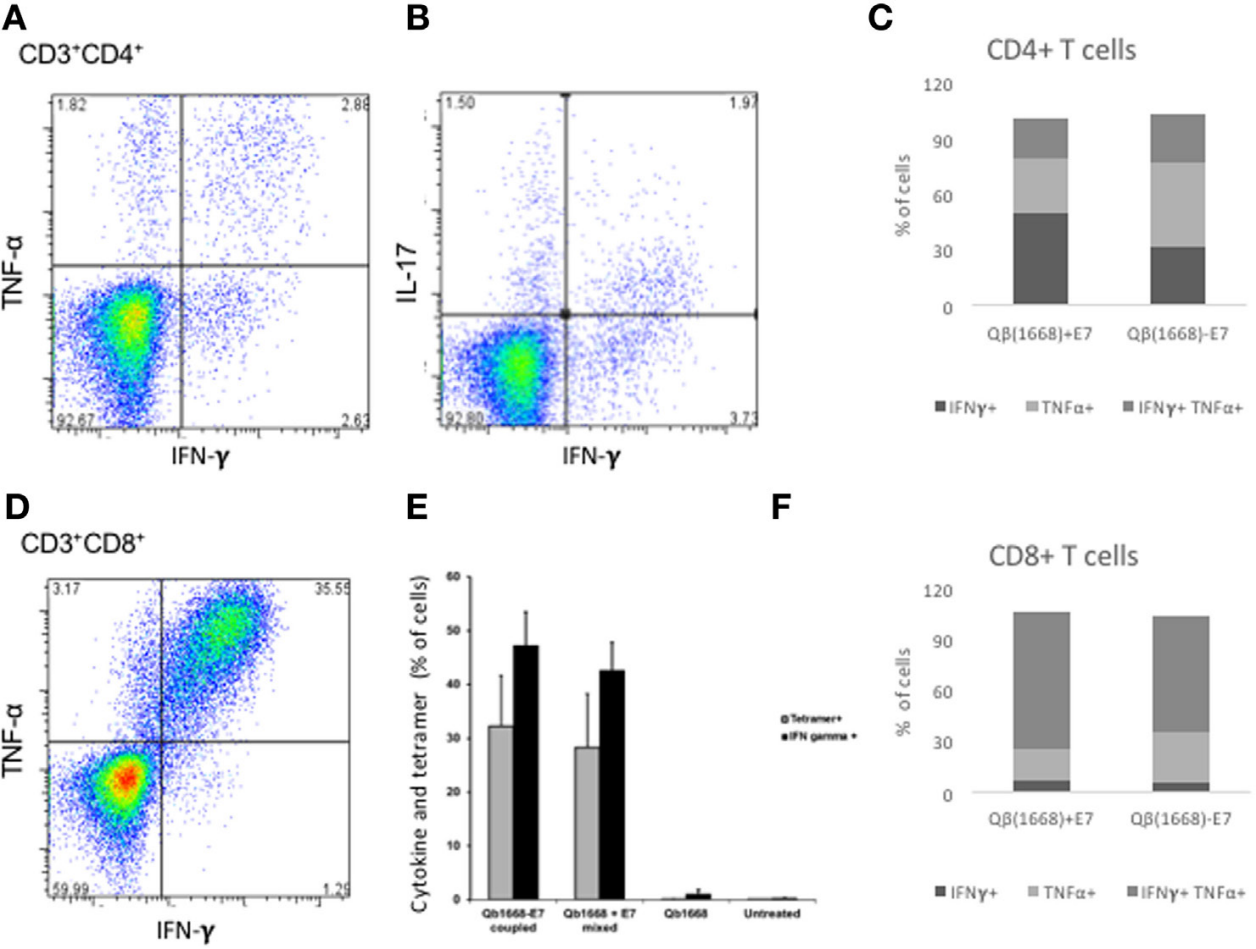
**Coupling of similar size antigens to VLPs is not required for cytokine production by T cells**. **(A)** TNFα and TNFγ production by CD4^+^ T cells upon vaccination with Qβ(1668) − E7. **(B)** IL-17 and TNFγ production by CD4^+^ T cells upon vaccination with Qβ(1668) − E7. **(C)** Cytokine production by CD4^+^ T cells upon vaccination with Qβ(1668) − E7. **(D)** Frequency of cytokine producing cells among CD8^+^ T cells upon *in vitro* stimulation. **(E)** Cytokine production by E7-tetramer specific CD8^+^ T cells. **(F)** Frequency of cytokine producing cells among CD4^+^ T cells upon *in vitro* stimulation.

To further investigate the cytokine production of splenic T cells, spleens were collected on day 78. Both vaccination regimens induced strong CD8^+^ and CD4^+^ T cell responses expressing multiple cytokines (Figure 5) and induction of multifunctional CD4^+^ T cells producing IFNγ, TNFα, and IL-17 (Figure 5A,B) were observed. For CD8^+^ T cells, a strong induction of TNFα and IFNγ-producing T cells was detected (Figure 5D,E). The number of tetramer-positive cells was roughly similar to the frequency of IFNγ-producing CD8^+^ T cells (Figure 5E). Thus, as observed in the blood, linked E7 protein induced similar frequencies of cytokine-producing T cells compared to the mixed formulation in splenic T cells (Figure 5).

**Figure 6:**
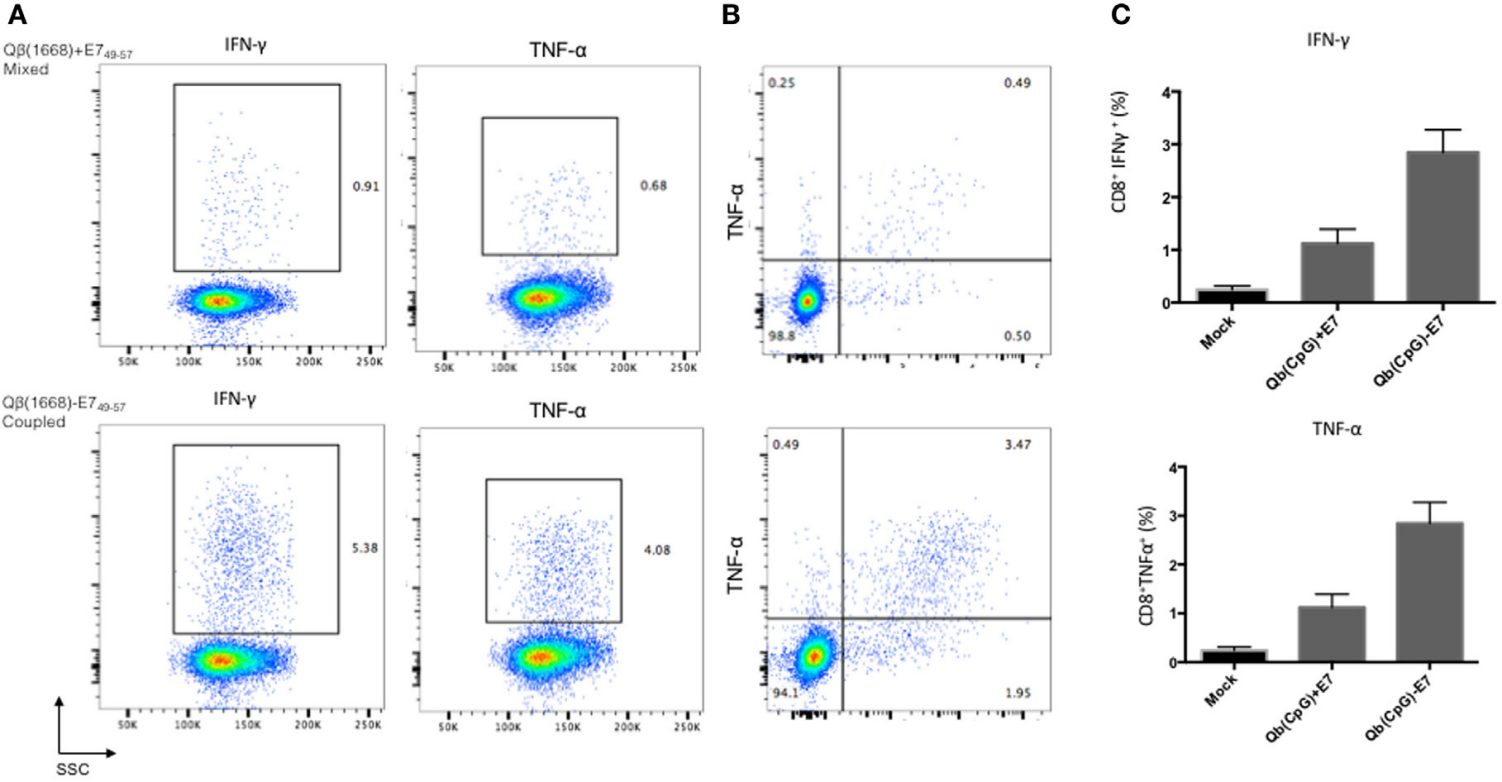
**Coupling of small peptides and VLP is required for activation of CD8^+^ T cells**. C57BL/6 female mice were immunized twice (days 0 and 7) s.c. with 50 μg of coupled or mixed Qβ(1668) and E7_49–57_. Spleens were harvested on day 14 for cytokine production. Cells were stimulated for 6 h with peptide and analysed by flow cytometry. **(A)** IFN-γ and TNF-α production by CD8^+^ T cells upon prime-boost vaccination with mixed (upper panel) and coupled (lower panel) versions of Qβ(1668) vaccine. **(B)** Coupled vaccine (bottom) induces multifunctional CD8^+^ T cells but not mixed (upper). **(C)** Percentage of CD8^+^ T cell producing IFNγ and TNFα post in vitro stimulation. Data represented as mean plus SEM, *n* = 5. Cells analyzed by FCM, 1 × 10^7^ cells acquired per sample. Values are plotted after subtraction of background values from non-stimulated samples.

In marked contrast to the results obtained with particulate E7 proteins, for small peptides, linkage to the VLP was essential. Specifically, mice immunized with the peptide E7_49–57_ coupled to Qβ(1668) generated strong CD8^+^ T cell responses. In contrast, E7_49–57_ mixed to Qβ(1668) failed to induce good production of IFNγ and TNFα by CD8^+^ T cells significantly above background (Figure 6).

### Mixing of E7 Protein with Qβ (1668) Is Sufficient for Eradication of Established Tumors

**Figure 7:**
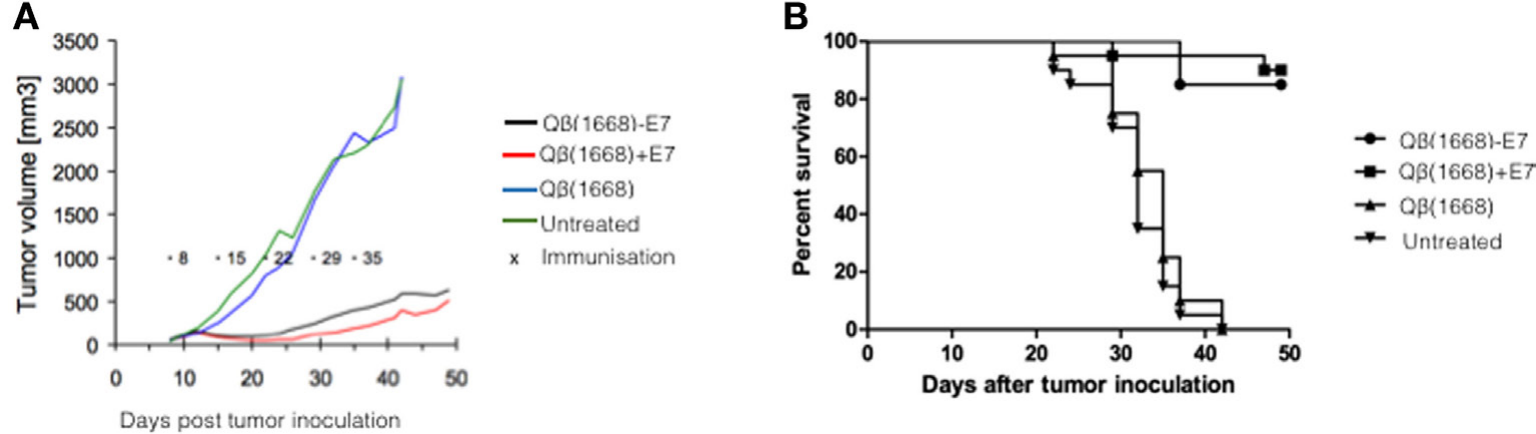
**Simply mixing of similar size antigen and adjuvant is enough for protection against tumor**. **(A)** Tumor growth after tumor challenge in immunized mice with mixed and coupled vaccines or controls was monitored over 50 posttumor inoculation. **(B)** C57BL/6 female mice (*n* = 5 per group) were injected i.v. with 1.5 × 10^5^ TC-1 cells expressing HPV-16 E7 oncoprotein. Crosses indicate the weekly immunization schedule with E7 coupled or mixed to Qβ(1668) for 49 days. **(B)** Percent of survival and tumor growth in immunized groups was monitored over time and represented in a Kaplan–Meier survival curve and analysed using the Log-rank test; *P < 0.05.

In order to assess the therapeutic capacity of the vaccine-induced T cells, mice were injected with TC-1 cells expressing HPV16 E7 oncoprotein. Eight days post injection of tumor cells, mice developed palpable tumors and were subjected to a weekly immunization schedule with E7 coupled or mixed to Qβ(1668) for 49 days (Figure 7A). In untreated mice, as well as mice injected with Qβ(1668) only, tumors grew unrestrained and 50% of mice reached severity scores requiring euthanasia by day 32 (Figure 7B). In contrast, both coupled and mixed formulation of Qβ(1668) and E7 protein were able to induce strong protection against tumor growth resulting in survival rate of more than 80% until the end of the study. In an additional study, vaccination with E7 mixed with Qβ(1668) extended the survival of tumor bearing mice to more than 3 months (data not shown). Therefore, covalent linkage of the E7 to Qβ(1668) VLPs is not required for induction of protective T cell responses against solid tumor.

### Covalent Linkage of E7 Oligomers to CpG-Loaded VLPs Is Required for Induction of Optimal B Cell Responses

**Figure 8:**
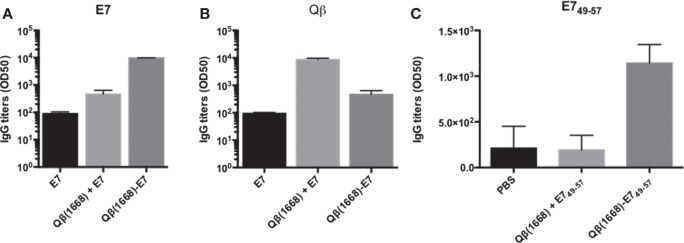
**Covalent linkage is required for optimal responses of B cells**. **(A)** Antibody titters for IgG against E7 expressed in log scale of OD50 on day 71. **(B)** Total IgG titters expressed in log scale of OD50 against Qβ at day 71 measured by ELISA. Mean with SEM in log transformed titters. **(C)** Antibody titters expressed in OD50 against the peptide E7_49–57_. Values as mean (*n* = 5) plus SEM.

In order to understand whether the same rules would apply to B cells, the importance of covalent linkage of E7 to Qβ VLPs for induction of optimal antibody responses was assessed. Serum collected from mice immunized with E7 coupled or mixed with Qβ(1668) were analyzed for E7-specific antibody response by ELISA. Importantly, only covalent coupling but not simple mixing of E7 and Qβ (1668) was able to enhance the antibody response against E7 (Figure 8A). Conversely, the antibody levels raised against Qβ were higher in the mixed formulation when compared to the coupled counterpart (Figure 8B). Antibody induction against the E7_49–57_ peptide also required covalent linkage, as in the absence of linkage, antibody levels induced were comparable to the background (Figure 8C). Hence, in contrast to T cell responses, B cell responses appear to require physical linkage of antigen and adjuvants in order to induce appropriate levels of antibody responses irrespective of the size of the antigen.

## Discussion

In the current study, we demonstrated in an HPV model that a particulate VLP-based vaccine containing CpG exerts distinct adjuvant effects in B and T cells. While for T cells, the concomitant draining of antigen and adjuvant to LNs and subsequent uptake by APCs is enough for T cell-mediated cytokine production and protection against the expansion of tumors, for B cells, appropriate presentation of antigens by linkage to VLPs is required for induction of optimal antibody levels.

This dichotomy may be explained by mechanistic differences in the induction of innate and adaptive immune responses. The innate arm of the immune system represented by DCs is able to recognize certain pathogen and danger-associated patterns (23). However, specific receptor–ligand interaction is not required in order to induce phagocytosis and activation (24). Thus, DCs will non-specifically take up both antigen and VLP. This is strictly different for B cells, which take up particles in an antigen-specific fashion. For B cells to be exposed to CpGs packaged within VLPs, they need to take up the particles via their B cell receptors (BCR), which will be followed by internalization of the VLP plus their CpG cargo (25). Therefore, E7-specific B cells will fail to take up VLPs loaded with CpGs unless E7 is linked to the VLPs. In addition to that, VLPs are potent activators of B cells (26, 27) and the presentation of the coupled antigens in the surface of the VLPs in a geometrically defined and repetitive manner promotes cross-linking of the BCR, which helps surpass the threshold for cellular activation (28). In the case of large antigens such as E7, the coupling is limited by steric hindrance of the proteins, limiting the number of copies of E7 that can be exposed. However, our data suggest that even with relatively low valency of the antigen, coupling exerts a positive impact on the level of antibodies produced.

For priming of T cell responses, activation of the DCs presenting the specific antigen is required (29). The simultaneous uptake of antigen and adjuvants by DCs, but not by T cells is critical (30). However, in contrast to B cells, DCs take up particulate antigens non-specifically without need for antigen-specific receptors (31). Given that DCs on average can accommodate and take up 70 particles (at a size of about 30 nm) (32), most DCs will take up reasonable numbers of both particle species at the same time. Therefore, simple co-exposure of DCs to particulate antigen and adjuvants is sufficient for simultaneous uptake of both entities. This guarantees that DCs will be appropriately activated providing optimal signaling to T cells.

Of particular note, the conclusions from this study are in agreement with a growing body of evidence that proposes that the size of antigens conveys major influence on factors driving immunity (14, 15, 17, 18). Peptides are well known to be unable to induce relevant immune responses; this is likely a result of inefficient drainage, degradation, or uptake by resident DCs *in vivo* that do not migrate to the LNs. A position that appears to have been confirmed here, as administered peptides failed to charge LN resident DCs.

Collectively, these observations are important for vaccine development, as they elucidate distinct requirements for induction of optimal B versus T cell responses and indicate that the use of both particulate antigens and adjuvants with similar size avoids the necessity of conjugating the two entities together. This may be especially important for the development of patient-specific vaccines. VLPs are an attractive platform for personalized vaccines considering the convenience of production and low costs.

Based on the findings of this work, a new advantage is attributed to such nanoparticles, as we show that covalent linkage of antigen and adjuvants might not be necessary to obtain protective tumor-specific T cells. This knowledge can be used to design antigens that would not require coupling, which would streamline the manufacturing process and increase cost-savings. Also, this simple system of mixed and coupled vaccines stands as an attractive tool to explore the dynamic of humoral and cellular responses and how they interact.

## Ethics Statement

This study was carried out in accordance with the recommendations of the Animals (Scientific Procedures) Act 1986 (ASPA) and European Directive 2010/63/EU on the protection of animals used for scientific purposes.

## Author Contributions

AG, AF, and FZ performed the experiments. MB, VM, and PS designed the experiments. AG, VM, and MB wrote the manuscript. GC-M and AT provided technical support.

## Conflict of Interest Statement

MB declares to be involved in a number of companies developing VLP-based vaccines. FZ is an employee of Hypopet AG. The other authors declare no further conflicts of interest.
